# The Epistemology of Evolutionary Psychology Offers a Rapprochement to Cultural Psychology

**DOI:** 10.3389/fpsyg.2020.579578

**Published:** 2020-11-03

**Authors:** Gad Saad

**Affiliations:** John Molson School of Business, Concordia University, Montreal, QC, Canada

**Keywords:** culture, epistemology, evolutionary psychology, sequential sampling, nomological networks of cumulative evidence, toy preferences, hourglass figure, consilience

## Abstract

Many detractors of evolutionary psychology (EP) presume that adaptive arguments are nothing more than whimsical and unfalsifiable just-so stories. The reality though is that the epistemology of EP is precisely the opposite of this antiquated canard in that it fixes the evidentiary threshold much higher than is typically achieved by most scientists. EP amasses evidence across cultures, time periods, disciplines, paradigms, methodologies, and units of analyses in validating a given scientific explanation. These nomological networks of cumulative evidence stimulate greater interdisciplinarity, lesser methodological myopia, and increased consilience (unity of knowledge). A component in building such nomological networks is to examine phenomena that are cross-culturally invariant (human universals) versus those that vary cross-culturally as adaptive responses (the domain of behavioral ecologists and gene-culture coevolution modelers). The epistemological efficacy of this unique approach is highlighted using two cases studies, the sex-specificity of toy preferences and men’s preference for the hourglass figure.

## Introduction

Many critics of evolutionary psychology (EP) wrongly presume that the field is largely focused on identifying human universals while ignoring cross-cultural differences (Saad, 2011, p. 27, claim 4). This speaks to the fact that many social scientists remain bound to the false nature-nurture dichotomy while evolutionary behavioral scientists have long recognized that culture does not exist outside of our biological heritage. As explained by the famed biologist Wilson (1978, p. 167), “The genes hold culture on a leash. The leash is very long, but inevitably values will be constrained in accordance with their effects on the human gene pool.” The cataloguing of human universals as well as the identification of innumerable cultural differences are within the purview of evolutionary theorizing. I demonstrate this crucial point by highlighting the manner by which cultural expressions (including human universals) are often necessary elements when building a nomological network of cumulative evidence in support of an evolutionary argument. A central feature of any such endeavor is knowing when one has amassed a sufficient level of cumulative evidence, a process captured by sequential sampling, and a topic to which I turn next.

## Sequential Sampling as an Epistemological Tool

Suppose that you are facing a choice between two cars to purchase, two politicians to vote for, or two prospective mates to go out with. How do you know when you have sampled enough information about the competing alternatives to make a final choice? In other words, when should you stop acquiring additional information about the alternatives, and choose a winning option accordingly? The stopping decision is a central element of human decision making notwithstanding the fact that classical economists would posit that individuals ought to acquire all of the relevant information prior to making a choice (as this would ensure that utility maximization is achieved). This form of sequential sampling was famously developed by Wald (1947) in the context of hypothesis testing. Specifically, the idea was that the sample size for a given experiment was not fixed but rather was determined based on when the iteratively collected data reached an absorption barrier (also known as a stopping threshold). For another early application of sequential sampling, imagine that you are a firm trying to establish whether your manufacturing process surpasses some benchmark of quality control (e.g., some minimal number of defective products are produced in the production line). How many products do you need to sample prior to establishing that you now have a sufficient amount of evidence to clear the manufacturing process?

**Figure 1:**
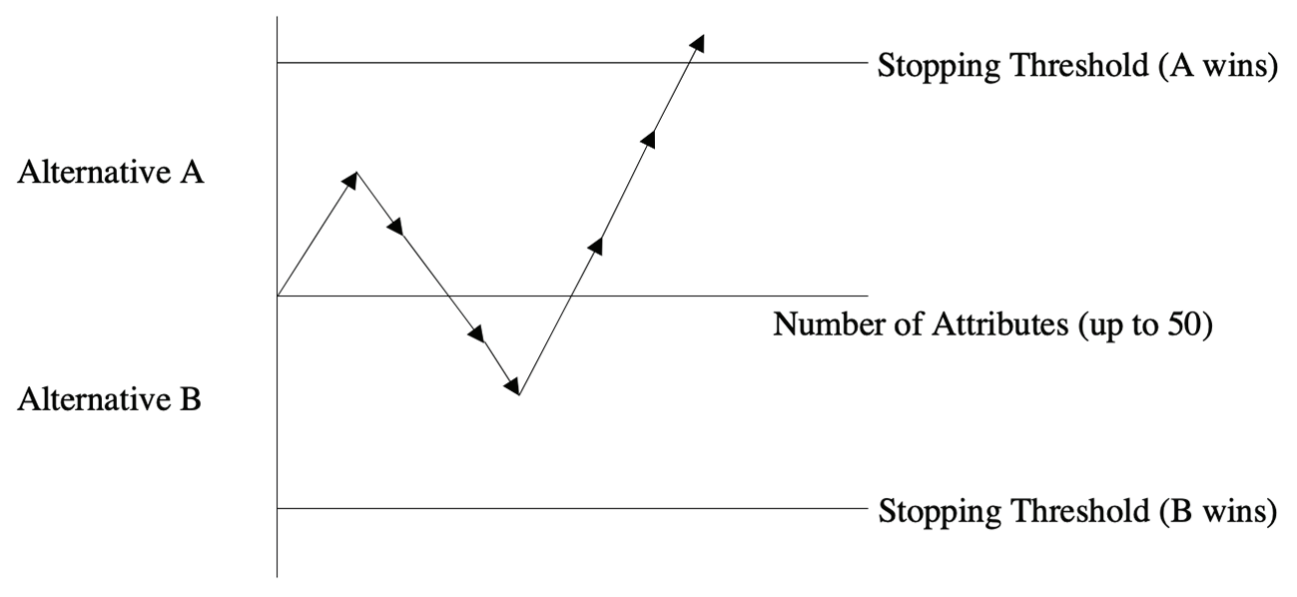
The binary sequential choice model.

This class of sequential sampling was eventually applied to binary multi-attribute choices (Aschenbrenner et al., 1984; Saad, 1994; Saad and Russo, 1996; Saad et al., 2009). In Figure 1, a pictorial representation of the process is shown. A decision maker is facing a choice between Alternatives A and B and must decide how many pieces of attribute information she should acquire on both alternatives prior to stopping and choosing a winning alternative. The stopping thresholds dictate the cumulative differentiation that must be achieved (as set by the decision maker in question) to be sufficiently convinced to stop the search process and choose the winning alternative (the one for which the stopping threshold is reached first). In the depicted example, the first piece of attribute information is in favor of Alternative A but since the threshold is not reached, the process continues. The second piece of attribute information is in favor of Alternative B but cumulatively speaking, A is still ahead (after two pieces of acquired attributes). After attributes 3 and 4 are acquired, B is clearly in the lead. However, once the fifth, sixth, and seventh attributes are acquired, the stopping threshold of A is surpassed causing the decision maker to stop acquiring additional information and choosing A accordingly.

Let us suppose that this hypothetical choice between A and B had up to 50 attributes that could have been acquired. The shown example demonstrates that the stopping threshold for A was reached after only 14% of the available information (seven out of 50 attributes) was acquired.

**Figure 2:**
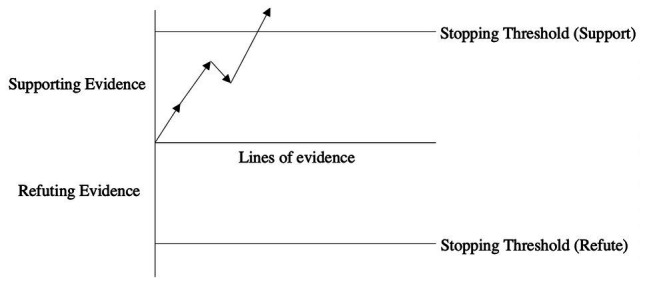
Sequential sampling of evidence for and against a scientific explanation.

This sequential model is applicable in a very broad range of contexts including in understanding a fundamental feature of the epistemology of science (Saad, 2017). How do we know when we have amassed a sufficient amount of evidence to suggest that a documented phenomenon can be incorporated into a field’s core knowledge (while recognizing that scientific knowledge is always provisional)? A literature review allows us to qualitatively gauge the extent of support for a given effect while a meta-analysis permits us to do so quantitatively. But while such approaches are very valuable in establishing the veracity of the standard proximate effects in science (say the strength and directionality of the causal relationship between variables X and Y), they are incapable of evaluating the evidentiary state of a grand theory. Take for example Charles Darwin’s *On the Origin of Species*. In order to build a persuasive case for his theory, Darwin amassed evidence from a very broad range of disciplines. These disparate sources of data all pointed to the same incontrovertible conclusion, namely that his theory was airtight. To place Darwin’s theory of evolution within the parlance of the current sequential model, he amassed enough confirming cumulative evidence to surpass a stopping threshold of support for his grand theory. In Figure 2, the first two lines of evidence offer strong support for the phenomenon under investigation while the third line of evidence provides some smaller measure of refutation. Finally, once the fourth line of evidence is presented, the support threshold is surpassed suggesting that a sufficient amount of evidence has been amassed to reach an epistemological conclusion. The length of each arrow captures the amount of cumulative support or refutation for a given line of evidence.

## Nomological Networks of Cumulative Evidence

**Figure 3:**
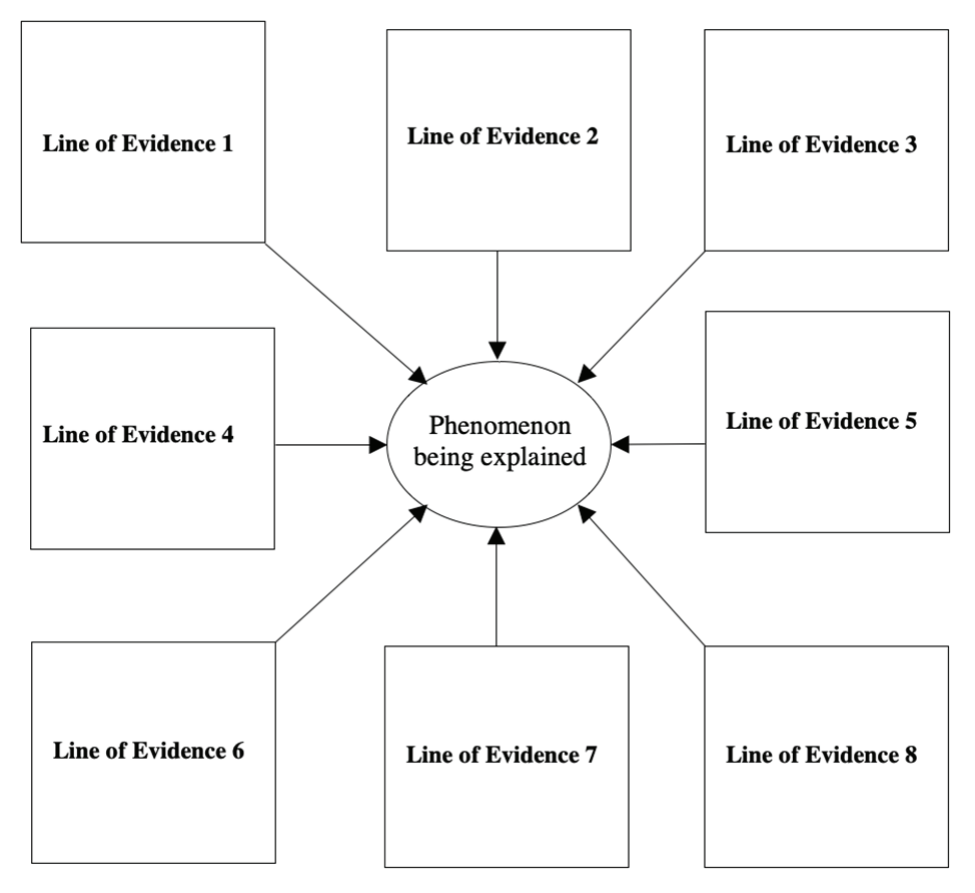
Nomological network with eight distinct lines of cumulative evidence.

This synthetic approach has been systematized via the use of nomological networks of cumulative evidence (Schmitt and Pilcher, 2004; Saad, 2017, 2020a, chapter 7; Saad, 2020b). The objective is to identify sources of data across disciplines (e.g., from cultural psychology, comparative psychology, developmental psychology, classics, behavioral genetics, medicine, and cognitive neuroscience), methodologies (surveys, archival data, lab and field experiments, and observational studies), measurements (e.g., paper-and-pencil, eye-tracking, hormonal markers, and fMRI data), cultures (e.g., establishing a human universal), and time periods (demonstrating the robustness of the phenomenon across eras) that converge onto an unassailable support for a given scientific explanation (see Figure 3 for a pictorial representation).

In a manner similar to how prosecutors seek to systematically build an inculpatory mountain of evidence that surpasses the “reasonable doubt” threshold, the building of a nomological network of cumulative evidence is meant to offer such incontrovertible support in the scientific domain. I shall demonstrate how this process works via two specific examples, both of which are poignant in that they have straddled the nature-nurture (or genes vs. culture) debate, namely the biological roots of toy preferences and the evolutionary basis of men’s preference for the female hourglass form (for a pictorial representation of these nomological networks, refer to Saad, 2017, Figures 2, 3).

## Toy Preferences: Socially Constructed or Biological Based?

Social constructivists typically argue that most sex differences arise due to differential socialization (cf. Eagly and Wood, 2012; Fausto-Sterling et al., 2012). When it comes to toy preferences, children are supposedly taught the appropriate sex-specific toys that they should prefer (e.g., truck vs. doll), and the sex-appropriate play styles that they should adopt (rough and tumble play for boys). These early lessons then apparently serve as the starting point of a never-ending cascade of gender role socialization that shapes the behavioral trajectory of the two sexes. How would one go about constructing a nomological network that casts doubts on the premise that sex-specific toy preferences are socially constructed?

A powerful way to demonstrate that a preference is not due to socialization is to highlight its presence in infants that have yet to reach the developmental stage to be socialized (think back of the cognitive stages delineated by Jean Piaget). For example, infants exhibit a preference toward attractive faces at an age when they could not have been socialized about the “social construction of beauty” (Langlois et al., 1990; Slater et al., 1998). Similarly, infants exhibit sex-specific toy preferences that rules out the socialization premise (Alexander et al., 2009; Jadva et al., 2010). Hence, one source of data in building the relevant nomological network stems from developmental psychology. Pediatric endocrinology offers another distinct line of evidence that also utilizes children samples. Girls diagnosed with congenital adrenal hyperplasia, a masculinizing endocrinological condition, hold more masculinized toy preferences than girls unafflicted with this condition (Berenbaum and Hines, 1992; Nordenström et al., 2002).

Hormonal effects on toy preferences were also examined using children stemming from non-clinical populations. The digit ratio (e.g., the 2D:4D digit ratio measures the relative lengths of the index and ring fingers) is a sexually dimorphic morphological marker that captures the extent to which individuals have been exposed to testosterone in utero. In pre-school boys, a negative correlation was documented between their digit ratios and their Pre-School Activities Inventory scores (PSAI; includes items that measure the extent to which children play with various types of toys) as collected from their parents (Hönekopp and Thierfelder, 2009). Smaller digit ratios correspond to greater masculinization while more positive PSAI scores signify more masculine play. In other words, boys who have more masculinized morphological features are more likely to engage in masculine play. Lamminmäki et al. (2012) assessed the testosterone levels of boys and girls starting at 7 days old until 6 months of age via monthly urinary samples. These were subsequently correlated to the infants’ PSAI scores and to their observed play behaviors with masculine, feminine, and gender-neutral toys at 14 months of age. The pattern of play with trains and dolls yielded the expected sex difference. Furthermore, PSAI scores and testosterone were positively correlated in boys, and the extent of play with the doll was negatively correlated with testosterone in boys while the extent of play with the train was positively correlated with testosterone for girls.

Comparative psychology serves as another powerful distinct line of evidence against explanations rooted in social constructivism. Specifically, if a phenomenon exhibits homologous regularity across several species, it likely possesses a biological/evolutionary signature. By demonstrating that infants of several primate species (vervet and rhesus monkeys and chimpanzees) exhibit sex-specific toy preferences congruent with those of human infants (Alexander and Hines, 2002; Hassett et al., 2008; Kahlenberg and Wrangham, 2010), this casts further doubt that toy preferences are socially constructed. The evidence presented thus far stemming from developmental psychology, pediatric endocrinology (clinical and non-clinical samples), and comparative psychology is more than sufficient to surpass the support threshold that toy preferences are not solely socially constructed (refer back to Figure 2). However, a compelling nomological network of cumulative evidence should be exhaustive if it is to prove maximally persuasive against all prospective detractors. With that in mind, several additional distinct lines of evidence are presented next.

If one wishes to argue that a given phenomenon is rooted in evolutionary and/or biological realities, it is often necessary to demonstrate that it is a human universal (albeit I will later explain how cross-cultural differences are also within the purview of the evolutionary lens). Readers interested in human universals in general and psychological universals in particular should refer to Brown (1991) and Norenzayan and Heine (2005), respectively. In a recent meta-analysis, Todd et al. (2018, p. 25) conclude “…the finding of robust sex differences in boys’ and girls’ toy preferences across a range of ages, different time periods, countries, and settings indicates an innate influence on this behavior…” However they note (p. 22) that “Despite the relative consistency across regions showing some support for biological determinants of preferences, a limitation of this meta-analysis, and of this research area in general, is that most of the research has been conducted in Western countries and findings cannot be generalized elsewhere.” In their meta-analysis, they included data from the Canada, the United States, Europe, Israel, and Hong Kong. However, as warned by Henrich et al. (2010), researchers must be mindful of not succumbing to a form of convenience sampling, namely, relying on samples that solely stem from Western, Educated, Industrialized, Rich, and Democratic (WEIRD bias) societies. With that in mind, Rossie (2005) conducted a comprehensive non-Western anthropological study of dolls and doll play stemming from a very broad group of peoples from the Saharan and North African regions who have maintained links to their ancestral heritage. Two key findings were that female dolls are much more prevalent than male dolls and playing with dolls is a domain largely reserved for girls. Of note, the universality of sex-specific toy preferences has been documented not only across cultures but also across epochs. An examination of funerary monuments from ancient Greece found that girls were shown with dolls, whereas boys were represented with wheels (Grossman, 2007, p. 321). It would appear that the sex-specificity of toy preferences is not only invariant across Western cultures but also applies in radically different cultural settings and time periods.

Of 50 countries whose masculinity-femininity scores have been tabulated, Sweden scores as the most feminine and the nation with the greatest gender equality (Hofstede, 1998, p. 9). As a matter of public policy, it has sought to systematically eradicate gender markers where possible (e.g., linguistic markers of gender), but more generally it has pursued a far-ranging social experiment meant to create a post-gender society. If there ever were a country in which sex-specific toy preferences might be lessened if not eradicated, it would be in Sweden, a truly ideal longitudinal experiment to gauge the veracity of the premise that toy preferences are socially constructed. Nelson (2005) took an inventory of actual toys that were present in the rooms of Swedish boys and girls aged 3 and 5 years of age. A total of 40,673 and 40,891 toys were inventoried in girls’ and boys’ rooms, respectively. The sex-specificity of toy preferences was no different than those repeatedly documented in other cultural settings. Apparently, no amount of social engineering can alter a cross-culturally invariant sexual dimorphism. Of note, the gender equality paradox, which refers to a broad range of phenomena beyond toy preferences, highlights the fact that countries with greater gender equality often exhibit the same if not *more* pronounced sex differences (cf. Costa et al., 2001 within the personality domain; Stoet and Geary, 2018 within the educational realm, and Zhang et al., 2019 for mating preferences).

Incidentally, to argue that the sex-specificity of toy preferences is innately determined does not negate the possibility that parents do indeed socialize their children to adopt such preferences. Patterns of socialization are not antithetical to evolutionary explanations rather they serve to reinforce biological imperatives. In a study of actual toy purchases, parents were intercepted upon leaving a toy store and queried about the purchases that they had just made and to whom the toys in question were targeted (Fisher-Thompson, 1993). These were coded as either sex-typed (i.e., sex appropriate) or sex-inappropriate. Only 1 and 3% of purchased toys were sex-inappropriate in studies 1 and 2, respectively. Evolutionary psychologists do not contest the fact that socialization occurs but to the extent that universally similar forms of parental influence take place, these are likely rooted in biological realities. More generally to state that “…culture is a partner in producing body systems commonly referred to as biology…” (Fausto-Sterling, 2005, p. 1516) overstates the role of culture in producing biological systems (including the human mind). Nurture exists in its varied forms because of nature albeit this in no way negates the fact that both are crucially important in defining our personhoods.

## The Hourglass Figure: Socially Constructed Beauty Marker or Evolved Preference?

Social constructivists have long argued that beauty is socially constructed. To some extent this is true in that some beauty markers and/or beauty rituals are culturally specific such as neck elongation (Padaung and Kareni women in Myanmar), lip plating (Surma women in Ethiopia), and foot binding (Chinese women). Contrary to most instances around the world where women are judged in beauty pageants, the Geerewol festival in West Africa involves Wodaabe men being the participants in an extravagant beauty pageant (a manifestation of a sex-role reversal). Each of the latter examples and many others point to the social construction of some beauty features and beauty rituals. However, it is also true that many beauty markers are universally operative. This is not surprising given that sexual selection, the evolutionary mechanism that selects for traits that bestows reproductive benefits, yields culturally invariant sex-specific mating preferences.

Let us consider men’s near-universal preference for the hourglass figure in women, which morphologically corresponds to a waist-to-hip ratio ranging from 0.68 to 0.72. The postulated reason for this preference, namely that it serves as an accurate signal of health and fertility, has been validated across several studies (see Singh, 2002 for relevant references but see Lassek and Gaulin, 2018a,b, and Bovet, 2019, for works that challenge this link). Cross-cultural preferences for the hourglass figure were obtained using photographs of women’s physiques prior to and subsequent to having had cosmetic surgeries, and via line drawings of women’s shapes exhibiting various waist-to-hip ratios (Singh et al., 2010 and references within). While many of the preferences were collected via paper and pencil tasks, more sophisticated methods have been employed including eye-tracking and brain imaging (Platek and Singh, 2010; Dixson et al., 2011). Content analyses have been conducted of female escorts’ online advertisements of their waist-to-hip ratios stemming from 48 countries across Europe, Asia, Oceania, Latin America, and North America (Saad, 2008), of Playboy centerfolds and Miss America winners across a number of decades (Singh, 1993), of 286 ancient Egyptian, African, Greco-Roman, and Indian sculptures and statuettes from several millennia ago (Singh, 2002), of 155 prehistoric Jomon figurines (Hudson and Ayoyama, 2006), and of Western artworks covering roughly 2,500 years (Bovet and Raymond, 2015). While some temporal variations do exist (consistent with the fact that the waist-to-hip preference is a near-universal), the general hourglass effect holds across these studies. The commercial premium that men place on the hourglass figure was captured by Griffith et al. (2016) in their analysis of online ads of female escorts. Specifically, online female escorts who advertise the hourglass figure charge higher fees. Finally, in a very compelling refutation of the premise that beauty standards are socially constructed (via visual media), congenitally blind men prefer the hourglass figure as elicited by touch (Karremans et al., 2010).

That the preference for the hourglass figure is a near-universal (rather than a human universal) recognizes the fact that cross-cultural differences do exist, and that these serve as adaptive responses to local ecological realities. For example, Marlowe and Wetsman (2001) demonstrated that in an ecosystem defined by lesser food surpluses, men’s preferences shift to a higher waist-to-hip ratio. On a related note, the prevalence of pathogens within a particular local niche has also been shown to affect the differential import ascribed to specific mate preferences, including physical attractiveness (Gangestad et al., 2006). In other words, specific mate preferences vary as a function of cultural contingencies. I turn to a discussion of this general issue next.

## Cultural Differences and Individual-Level Heterogeneity as Adaptive Responses

While the identification of human universals is a central feature of the evolutionary paradigm, cross-cultural differences as well as individual-level heterogeneity are also within its purview (Brown et al., 2011). Behavioral ecology, a sub-branch of the evolutionary behavioral sciences, is rooted in the fundamental understanding that humans exhibit wide behavioral plasticity as adaptive responses to local niches (Winterhalder and Smith, 2000; Nettle et al., 2013). In other words, the capacity to adapt is itself an adaptation akin to how the human immune system has evolved built-in flexibility permitting it to mount responses against novel and/or rapidly mutating pathogens (Cziko, 2000). This adaptive plasticity manifests itself in numerous ways including in the varied expressions of cultural forms. Take for example gastronomic traditions, which differ in endless ways across the globe. What determines the extent to which a culinary tradition will contain meat versus vegetable dishes? Why are some cuisines much spicier than others? Why do some cultures engage in geophagy (eating of dirt)? Why do certain food taboos arise? Most social scientists that tackle such issues do so as a means of documenting the rich and diverse forms of cultural expressions. Evolutionary scientists on the other hand ask the ultimate Darwinian *why*, namely, they seek to establish whether these culinary traditions might be adaptive responses to possible pathogenic exposure.

Billing and Sherman (1998) analyzed the frequency with which 43 different spices were used in meat-based cuisines of 36 countries across Africa, North America, Asia, Oceania, and Europe by examining 4,578 recipes from 93 cookbooks. Furthermore, they tabulated the yearly averages for temperature and precipitation (rain) for the countries in question. Since spices possess a wide range of antimicrobial properties, and given that food-borne pathogens are more prevalent in warmer and wetter climates, the researchers reasoned that spice use would correlate with these environmental conditions. This is precisely what they found. In a follow-up study using data from the same 36 countries, Sherman and Hash (2001) examined the use of 41 spices in 2,129 vegetable-only recipes from 107 traditional cookbooks. Their key objective was to establish that since food-borne pathogens are more prevalent in meat-based as compared to vegetable-only dishes, the latter should necessitate the lesser use of spices. This was strongly confirmed using both countries and spices as the unit of analysis. Similar antimicrobial principles have established the adaptive nature of anti-spoilage techniques in Japanese food (Ohtsubo, 2009), Fijian food taboos as a means of protecting against marine toxins (Henrich and Henrich, 2010), kosher prohibitions regarding shellfish (Saad, 2011), and geophagy or the eating of earth (Young et al., 2011).

I have thus far provided examples of food-related cultural traditions that serve as instantiations of adaptive processes. At a more fundamental level, some food-related adaptations are encoded in the human genome in ways that vary across cultures (cf. the Human Genome Diversity Project housed at Stanford University). For example, the global distribution of lactase persistent genes (help in the digestion of lactose) is correlated with pastoral living. This is a manifestation of gene-culture coevolution (Laland et al., 2010; Henrich, 2016; Moya and Henrich, 2016), the process by which a cultural practice (pastoral living) shapes the selection of genes that are advantageous within a given environment. Other diet-related cross-cultural differences in genetic expressions include those that conserve sodium in near-equatorial populations (Weder, 2007), as well those that help in the digestion of starch (Perry et al., 2007) and in the metabolism of alcohol (Osier et al., 2002). Viewed from this perspective, aggregate consumption patterns of particular foods and drinks are in part due to cross-cultural genetic differences that originally evolved as adaptations to local niches.

While it is perhaps consistent with people’s folk wisdom to link culinary traditions to pathogens, a growing number of studies have documented pathogenic effects in surprising contexts including in explaining country-level differences in scores on personality traits and cultural values. These include sociosexuality, openness to experience, and extraversion (Schaller and Murray, 2008), religiosity and the strength of family bonds (Fincher and Thornhill, 2012), conformity (Murray et al., 2011), and individualism-collectivism (Fincher et al., 2008). The general theoretical thesis is that aggregate-level personality scores and cultural traits (integral elements of a nation’s character) in part arise as adaptive responses to local niches (Nettle and Penke, 2010). Cultural neuroscientists are increasingly gathering empirical support for this premise at the genetic level. Specifically, cross-cultural distributions of several aggregate-level personality scores and cultural traits are correlated to the differential frequencies of genetic polymorphisms associated with the traits in question (Chen et al., 1999; Chiao and Blizinsky, 2010; Way and Lieberman, 2010).

## Conclusion

The epistemology of evolutionary psychology yields a rapprochement to cultural psychology in at least two distinct ways. First, when seeking to demonstrate the veracity of an evolutionary-based phenomenon, the corresponding nomological network of cumulative evidence will typically include at least one distinct line of evidence stemming from cultural psychology (establishing the universality of the phenomenon in question). Second, rather than the erroneous reflex of many social scientists to pit cultural and evolutionary explanations against one another, some cross-cultural differences are adaptive expressions across various units of analyses (e.g., genes, personality types, cultural traits, and cultural traditions such as local cuisines). The cataloguing of these adaptive responses could serve as one of the distinct lines of evidence in a nomological network (i.e., one of the boxes shown in Figure 3). Culture does not exist in a vacuum detached from our biological heritage. Rather, our common human nature and some of our cultural differences are instantiations of the long reach of evolution in shaping the rich tapestry of human expressions.

Nomological networks of cumulative evidence yield many important advantages irrespective of one’s research interests. One need not be an evolutionary psychologist to appreciate the epistemological benefits of this synthetic approach. Twenty years ago, Wilson (1999) spoke of the importance of seeking consilience across the social sciences, natural sciences, and the humanities. Nomological networks of cumulative evidence systematize this laudable objective by encouraging scholars to think of all possible sources of evidence that might test the veracity of their scientific explanations (Saad, 2017, 2019). If you refer back to the two case studies described earlier, the lines of evidence originate from across the social sciences, the natural sciences, and the humanities. E. O. Wilson’s call for consilience was heeded. Garcia et al. (2011) contrasted the number of disciplines represented by first authors of articles published in evolutionary-based psychology journals (e.g., *Evolution and Human Behavior*) to non-evolutionary-based ones (e.g., *Journal of Experimental Psychology: Learning, Memory, and Cognition*). Not surprisingly, they documented that the evolutionary perspective promotes greater interdisciplinarity. The building of nomological networks of cumulative evidence by definition accentuates the ethos of interdisciplinarity by forcing scholars to search assiduously for supporting as well as refuting evidence well outside their disciplinary silos. While most psychologists are constrained by methodological and field fixation (Sternberg and Grigorenko, 2001), nomological networks of cumulative evidence offer an opening toward methodological pluralism (free yourself of your experimental priming paradigm that you learned as a graduate student!). Finally, given the replication crisis in psychology that has been written about extensively, nomological networks amass cumulative evidence stemming from direct and conceptual replications (Schmidt, 2009) but more importantly they uncover what I coin as meta-replications (i.e., replications of a grand effect across a very broad range of disciplines, cultures, time periods, methodologies, etc.). Muthukrishna and Henrich (2019) argued that the replication crisis in psychology stems in part due to a lack of organizing theoretical frameworks. Nomological networks of cumulative evidence speak to this lacuna of theoretical consilience.

As an evolutionary behavioral scientist and a consumer psychologist, I have long been interested in the interplay between our shared biological heritage and our idiosyncratic cultural environments in shaping *Homo consumericus*. One of my prospective projects is to create a global consumer atlas that would identify universal consumer phenomena along with those shaped by cultural forces; a database akin to the Human Relations Area File (started at Yale University) or the Standard Cross-Cultural Sample albeit for consumer phenomena. Typically, consumer researchers have focused with much greater alacrity on identifying cross-cultural differences rather than human universals. This might be due to the misguided sense that to catalog similarities across cultures is akin to finding null effects, whereas cross-cultural differences are “significant” effects. I suspect that this bias is likely pervasive across other subdisciplines of psychology. That said, it is my hope that in the near future, scholars in general and psychologists in particular will be exposed to the synthetic power of nomological networks of cumulative evidence. Through the application of this formidable epistemological tool, cultural psychologists and evolutionary psychologists will likely experience a much-needed rapprochement.

## Author’s Note

GS is Professor of Marketing and former holder of the Concordia University Research Chair in Evolutionary Behavioral Sciences and Darwinian Consumption (2008–2018), John Molson School of Business, Concordia University.

## Author Contributions

The author confirms being the sole contributor of this work and has approved it for publication.

### Conflict of Interest

The author declares that the research was conducted in the absence of any commercial or financial relationships that could be construed as a potential conflict of interest.
